# Adipocyte Calpain‐2 Deficiency Reduces Obesity‐Accelerated Abdominal Aortic Aneurysm Formation in Mice

**DOI:** 10.1096/fba.2025-00202

**Published:** 2025-10-16

**Authors:** Ana Clara Frony, Aida Javidan, Weihua Jiang, Michihiro Okuyama, Lihua Yang, Haruhito A. Uchida, Venkateswaran Subramanian

**Affiliations:** ^1^ Division of Cardiovascular Medicine, Department of Medicine University of Missouri Columbia Missouri USA; ^2^ Saha Cardiovascular Research Center University of Kentucky Lexington Kentucky USA; ^3^ Division of Kidney, Diabetes and Endocrine Diseases Okayama University Medical Development Field Okayama Japan; ^4^ Department of Physiology University of Kentucky Lexington Kentucky USA; ^5^ Department of Medical Pharmacology and Physiology University of Missouri Columbia Missouri USA; ^6^ Dalton Cardiovascular Research Center University of Missouri Columbia Missouri USA; ^7^ Division of Cardiovascular Medicine, Department of Internal Medicine University of Nebraska Medical Center Omaha Nebraska USA

**Keywords:** abdominal aortic aneurysm, adipocytes, angiotensin II, calpain, obesity

## Abstract

Abdominal adiposity is associated with increased risk of abdominal aortic aneurysm (AAA) development. Calpains are non‐lysosomal calcium‐dependent cysteine proteases that are highly expressed in human and experimental AAAs. Using a pharmacological inhibitor and genetically deficient mice, we previously demonstrated that calpain‐2 (a major ubiquitous isoform) deficiency mitigated angiotensin II (AngII)‐induced AAA formation in hypercholesterolemic mice. In addition, we also demonstrated that calpain inhibition strongly suppressed adipose tissue inflammation in obese mice. Here, we evaluated the contribution of adipocyte‐specific calpain‐2 on obesity‐accelerated AAA in mice. Calpain‐2 protein is highly expressed in the periaortic adipose tissue (PAAT) of AngII‐induced AAAs in obese mice. To determine the relative contribution of calpain‐2 in obesity‐accelerated AAA development, calpain‐2 floxed mice were bred to mice with a tamoxifen‐inducible form of Cre under control of either the ubiquitous promoter, chicken β‐actin, or adipocyte‐specific promoter, Adipoq. Ubiquitous or adipocyte‐specific depletion of calpain‐2 in mice significantly suppressed Ang II–induced AAA formation in obese mice. In addition, calpain‐2 depletion reduced the incidence of AngII‐induced AAAs in mice. Furthermore, calpain‐2 deficiency prevented AngII‐induced aortic medial elastin fragmentation, adventitial collagen disruption, and periaortic leukocytic accumulation. These results suggest that adipocyte‐derived calpain‐2 plays a critical role in AngII‐induced AAA development in diet‐induced obese mice.

## Graphical abstract

Adipocyte‐specific deletion of calpain‐2 in mice decreases obesity‐accelerated abdominal aortic aneurysm formation, which is associated with reduced aortic medial elastin fragmentation and periaortic adipose tissue leukocyte accumulation. Targeted inhibition of calpain‐2 may offer a new therapeutic target to reduce obesity‐accelerated abdominal aortic aneurysms.

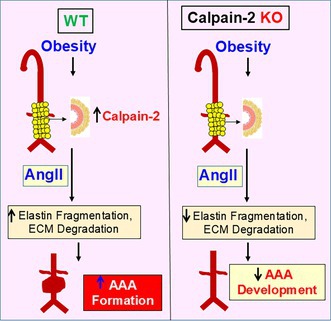

## 1 Introduction

Cardiovascular disease continues to be the primary cause of death in obese patients [1], and abdominal adiposity is strongly associated with increased prevalence of abdominal aortic aneurysm (AAA) in both humans and mice [2]. Recently, we demonstrated that AAA, a permanent dilation of the abdominal aorta, is strongly associated with increased calpain‐2 protease in both humans and non‐obese hypercholesterolemic mice [3]. Calpain‐2, a calcium‐dependent cysteine protease, is highly present in aortic adventitia and surrounding periaortic adipose tissue of human AAAs and angiotensin II (AngII)‐induced AAAs in non‐obese hypercholesterolemic mice [3]. In addition, we demonstrated that inducible depletion of calpain‐2 in vessel wall‐derived fibrogenic mesenchymal cells prevented AngII‐induced AAA formation in mice [3]. Furthermore, we showed that in diet‐induced obese mice, calpain inhibition by transgenic overexpression of its endogenous inhibitor, calpastatin, suppressed adipose tissue inflammation [4]. Based on our findings, we hypothesized that calpain‐2 in adipocytes is a contributor to obesity‐accelerated AAA formation in mice.

## 2 Methods

All animal experiments were approved by the University of Missouri (Protocol # 40503) and the University of Kentucky Institutional Animal Care and Use Committee (Protocols‐2011‐0907, 2020‐3634). Calpain‐2 floxed (f/f) mice were bred with transgenic mice expressing either tamoxifen‐inducible Cre recombinase under the control of the β‐actin promoter (Jackson lab; CAG‐Cre‐ER; stock#004682) or constitutive Cre recombinase under the control of the adiponectin promoter (Jackson lab; Adipoq‐Cre; stock#028020) to generate either inducible whole body or adipocyte‐specific calpain‐2–deficient mice [3]. Inducible Cre recombinase was activated by intraperitoneal injection of tamoxifen (25 mg/kg/day; C8267; Sigma‐Aldrich) for five consecutive days [3]. Obesity was induced by feeding a high‐fat diet (60% kcal diet; Research Diets D12492) for 16–20 weeks [4]. To induce AAA, diet‐induced‐obese mice were infused with AngII (1000 ng/kg/min) using osmotic mini‐pumps for 28 days [3]. For AAA studies, only male mice were used, as supported by the American Heart Association—ATVB Council 2018 recommendations for mice AAA studies [5]. Body weight, systolic blood pressure, body fat mass, glucose, and insulin tolerance tests were measured during the study, as described earlier [4]. After the end of the study, the maximum abdominal aortic diameter was measured ex vivo to quantify the AAA development [3]. Data were tested for normality and equal variance, and statistical analyses were performed using SigmaPlot 15.0 (SYSTAT Software Inc.).

## 3 Results

**FIGURE 1:**
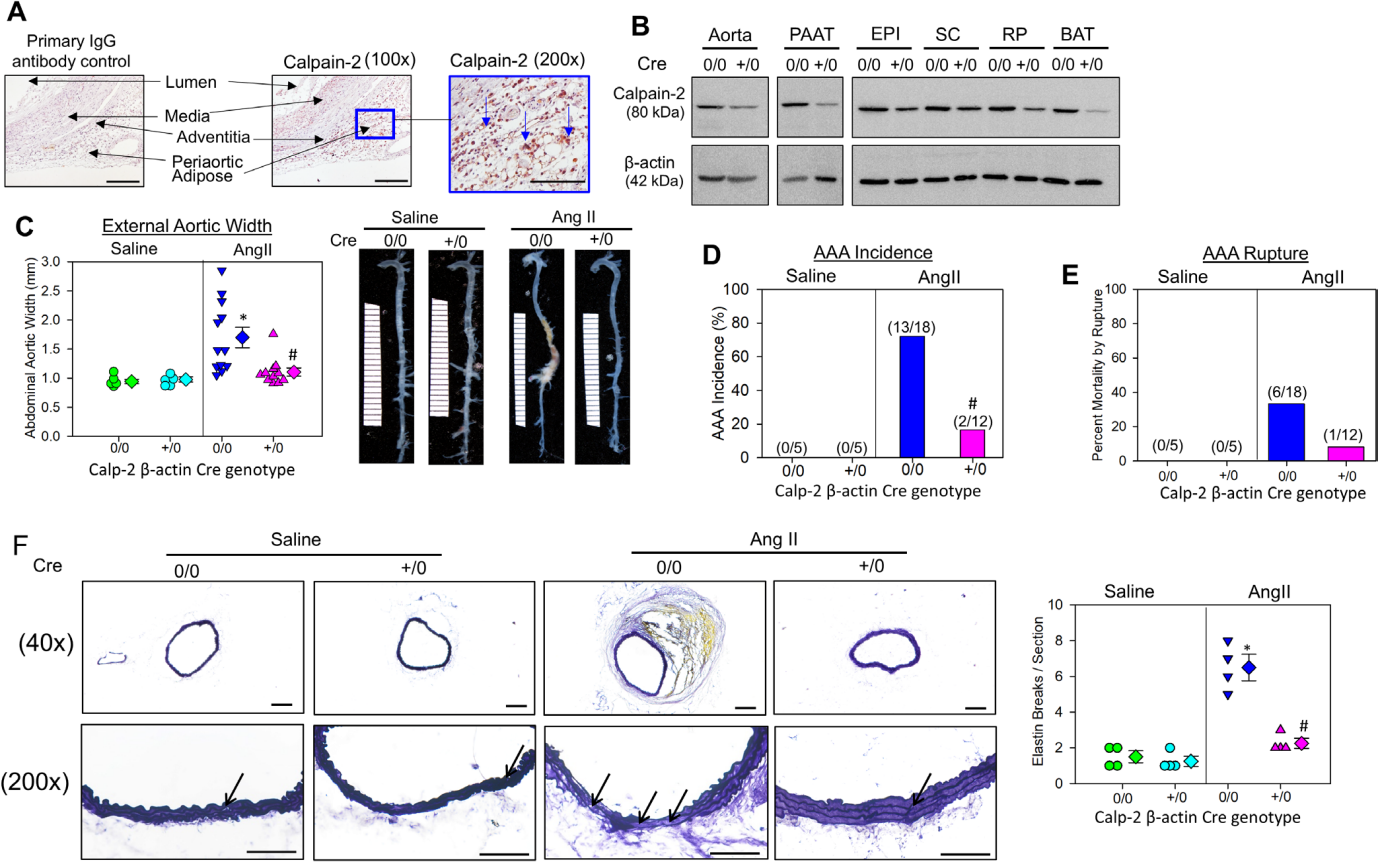
Calpain‐2 deficiency reduced obesity and accelerated AngII‐induced AAAs. (A) Aortic cross‐sections from AngII‐induced AAA tissue sections from obese mice were immunostained with anti‐calpain‐2 (10 μg/mL, catalog No. RP‐3 calpain‐2; Triple Point biologics, OR). Positive staining—reddish brown color highlighted with blue arrows. Scale bar corresponds to 50 μm (100× and 200× magnification). (B) Calpain‐2 and β‐actin protein in aorta, periaortic adipose tissue (PAAT), epididymal (EPI), subcutaneous (SC), retroperitoneal (RP), and brown adipose tissue (BAT) from calpain‐2 f/f β‐actin (ERT2) Cre 0/0 and +/0 mice. (C) Measurements of maximal external width of abdominal aortas (saline, *n* = 5; Ang II, *n* = 12–18). Green (saline—Cre 0/0), teal (saline—Cre +/0), blue (Ang II—Cre 0/0), and pink (Ang II—Cre +/0) represent individual mice, diamonds represent means, and bars are SEMs. Representative aortic images nearest the mean of each group. *p < 0.001 AngII versus saline; #*p* < 0.001 Cre +/0 AngII versus Cre 0/0 AngII; two‐way ANOVA followed by Holm–Sidak post hoc test. (D) and (E) the AAA incidence and mortality due to AAA rupture in AngII‐infused Cre 0/0 and Cre +/0 mice (#*p* < 0.05 Cre +/0 AngII vs. Cre 0/0 AngII; Fisher's exact test). (F) Representative images and quantification of AAA sections from Cre 0/0 and Cre +/0 mice infused with either saline or AngII stained for aortic medial elastin breakage (Verhoeff's staining; *N* = 4 *p < 0.05 AngII vs. saline, #*p* < 0.05 Cre +/0 AngII vs. Cre 0/0 AngII; Two‐way ANOVA followed by Holm–Sidak post hoc test).

Diet‐induced obesity is shown to accelerate AngII‐induced AAA formation in mice [6]. To examine the presence of calpain‐2 in the surrounding periaortic adipose tissue of AngII‐induced AAA in diet‐induced obese mice, 8‐week‐old C57BL6/J male mice were fed with a 60% Kcal diet (HFD; Research Diets D12492) for 16 weeks to develop obesity [4]. Immunohistochemical staining [3, 4] of abdominal aortic tissue with intact periaortic adipose tissue identified a strong distribution of calpain‐2 protein in the surrounding perivascular adipose tissue, with weak staining in the aortic media and intima (Figure 1A).

Since calpain‐2 deficiency in mice is embryonically lethal [7], to test our hypothesis, we generated whole body calpain‐2–deficient mice in a normolipidemic C57BL/6 background using calpain‐2 floxed (f/f) and tamoxifen‐inducible chicken β‐actin Cre promoter (ERT2 Cre+/0) mice as described [3]. Western blot analyses showed a strong reduction of calpain‐2 protein in aortas, periaortic adipose tissue (PAAT), and other fat pads—epididymal (EPI), subcutaneous (SC), retroperitoneal (RP), and brown adipose (BAT) from Cre+/0 mice (Figure 1B). Eight weeks old male Calpain‐2 f/f ER2 Cre+/0 and littermate control (Cre0/0) mice were fed with a 60% Kcal diet (HFD) for 20 weeks to develop obesity. After 16 weeks of diet feeding, mice were infused with either saline or AngII (1000 ng/kg/min) for 28 days [3] to induce AAA formation and continued on HFD. Inducible calpain‐2 deficiency did not affect HFD‐induced body weight (mean body weight; saline—Cre0/0: 58 ± 0.7 g vs. Cre+/0: 55 ± 1.2 g, AngII—Cre0/0: 49 ± 1.8 g vs. Cre+/0: 48 ± 1.1 g, *p* = NS; two‐way repeated measures ANOVA), fat mass (saline—Cre0/0: 24 ± 0.7 g vs. Cre+/0: 22 ± 1.3 g, AngII—Cre0/0: 20 ± 1.8 g vs. Cre+/0: 22 ± 0.9 g, *p* = NS; two‐way ANOVA), and lean mass (saline—Cre0/0: 26 ± 1.5 g vs. Cre+/0: 28 ± 0.7 g, AngII—Cre0/0: 25 ± 0.8 g vs. Cre+/0: 25 ± 0.8 g, *p* = NS; two‐way ANOVA) gain, glucose and insulin tolerance (data not shown), or AngII‐induced systolic blood pressure in obese mice (mean systolic pressure; saline—Cre0/0: 115 ± 3 vs. Cre+/0: 110 ± 2, *p* = NS; AngII—Cre0/0: 165 ± 6 vs. Cre+/0: 141 ± 5, *p* = NS; *p* < 0.05 saline vs. AngII; Two‐way ANOVA). Calpain‐2 deficiency significantly reduced AngII‐induced AAA formation (Figure 1C) as measured by external aortic width expansion [3] (mean width; saline—Cre0/0: 0.94 ± 0.04 mm vs. Cre+/0: 0.98 ± 0.04 mm, *p* = NS; AngII—Cre0/0: 1.7 ± 0.18 mm vs. Cre+/0: 1.1 ± 0.07 mm, *p* < 0.001; two‐way ANOVA). Furthermore, calpain‐2 deficiency also significantly reduced AngII‐induced AAA incidence (Figure 1D; defined as a > 50% increase in suprarenal aorta width; Cre0/0: 72% vs. Cre+/0: 7%, *p* < 0.001; Fisher's exact test) but had no significant effect on aortic rupture (Figure 1E, Cre0/0: 37% vs. Cre+/0: 8%). Verhoeff's staining of abdominal aortas revealed significant disruption of the focal medial elastin layer (Figure 1F) in the Cre0/0 group infused with AngII. However, the abdominal aortas from calpain‐2–deficient mice (Cre+/0) showed preserved medial elastin layer (Figure 1F) upon AngII infusion (elastin breaks; saline—Cre0/0: 1.5 ± 0.33 vs. Cre+/0: 1.25 ± 0.28, *p* = NS; AngII—Cre0/0: 6.5 ± 0.74 vs. Cre+/0: 2.25 ± 0.28, *p* < 0.05; Two‐way ANOVA).

**FIGURE 2:**
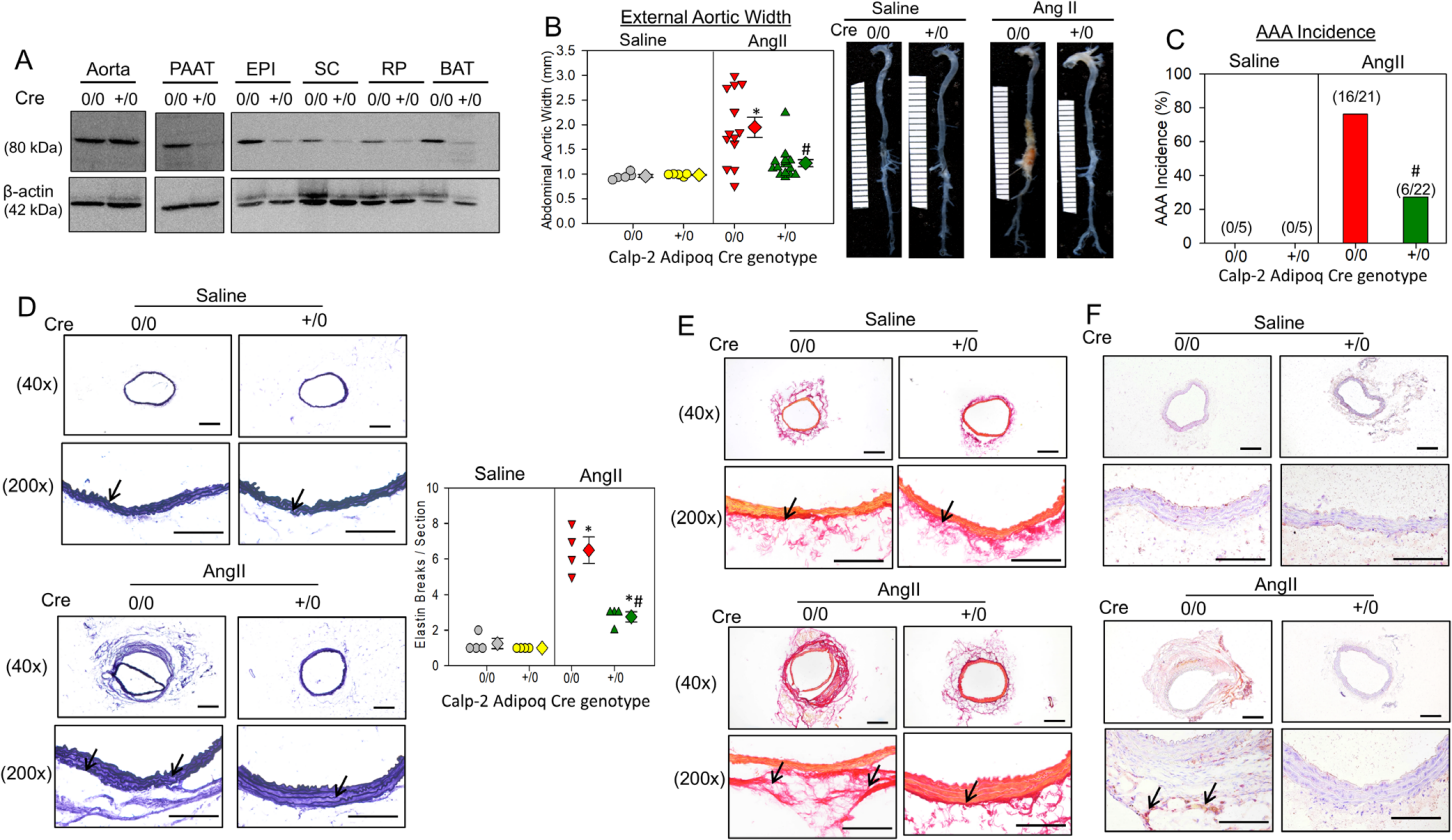
Adipocyte calpain‐2 deficiency reduced obesity and accelerated AngII‐induced AAAs. (A) Calpain‐2 and β‐actin protein in aorta, periaortic adipose tissue (PAAT), epididymal (EPI), subcutaneous (SC), retroperitoneal (RP), and brown adipose tissue (BAT) from calpain‐2 f/f Adipoq Cre 0/0 and +/0 mice. (B) Measurements of maximal external width of abdominal aortas (saline, *n* = 5; Ang II, *n* = 21–22). Gray (saline—Cre 0/0), yellow (saline—Cre +/0), red (Ang II—Cre 0/0), and green (Ang II—Cre +/0) represent individual mice, diamonds represent means, and bars are SEMs. Representative aortic images nearest the mean of each group. *p < 0.001 AngII versus saline; #*p* < 0.001 Cre +/0 AngII versus Cre 0/0 AngII; two‐way ANOVA followed by Holm–Sidak post hoc test. (C) AAA incidence in AngII‐infused Cre 0/0 and Cre +/0 mice (# *p* < 0.05 Cre +/0 AngII versus Cre 0/0 AngII; Fisher exact test). (D) Representative images and quantification of AAA sections from Cre 0/0 and Cre +/0 mice infused with either saline or AngII stained for aortic medial elastin breakage (Verhoeff's staining; *N* = 4 *p < 0.05 AngII vs. saline, #*p* < 0.05 Cre +/0 AngII vs. Cre 0/0 AngII; Two‐way ANOVA followed by Holm–Sidak post hoc test). (E) Representative images of AAA sections from Cre 0/0 and Cre +/0 mice infused with either saline or AngII stained for collagen (Picro Sirius red) and (F) leukocytes (anti‐CD45 antibody, 3 μg/mL, BD 553077, BD Pharmingen). Positive staining—reddish brown color highlighted with black arrows. Scale bar corresponds to 50 μm (40× and 200× magnifications).

To explore the functional role of adipocyte‐derived calpain‐2 in obesity‐accelerated AAA formation, we generated adipocyte‐specific calpain‐2–deficient mice using Adipoq Cre recombinase mice and calpain‐2 floxed mice as described [3]. Western blot analyses showed a strong reduction of calpain‐2 in various fat pads (PAAT, EPI, SC, RP, BAT) with intact calpain‐2 in the aorta (Figure 2A). After 16 weeks of diet feeding, mice were infused with either saline or AngII for 28 days and continued on HFD. As similar to whole body deficiency, adipocyte‐specific calpain‐2 deficiency did not affect HFD‐induced body weight (mean body weight; s.aline—Cre0/0: 50 ± 1.17 g vs. Cre+/0: 51 ± 1.1 g, AngII—Cre0/0: 41 ± 1.1 g vs. Cre+/0: 42 ± 1.2 g, *p* = NS; Two‐way Repeated Measures ANOVA), and fat mass (saline—Cre0/0: 17.5 ± 1.2 g vs. Cre+/0: 18.0 ± 0.8 g, AngII—Cre0/0: 17.5 ± 0.51 g vs. Cre+/0: 18.2 ± 0.42 g, *p* = NS; Two‐way ANOVA) and lean mass (saline—Cre0/0: 27.6 ± 1.3 g vs. Cre+/0: 28.2 ± 0.7 g, AngII—Cre0/0: 23.0 ± 0.43 g vs. Cre+/0: 24.7 ± 0.78 g, *p* = NS; Two‐way ANOVA) gain, glucose and insulin tolerance (data not shown), or AngII‐induced systolic blood pressure in obese mice (mean systolic pressure ‐mm/Hg; saline—Cre0/0: 125 ± 5 vs. Cre+/0: 111 ± 7, *p* = NS; AngII—Cre0/0: 170 ± 7 vs. Cre+/0: 167 ± 6, *p* = NS; *p* < 0.05 saline vs. AngII; Two‐way ANOVA). Calpain‐2 deficiency in adipocytes significantly reduced AngII‐induced AAA formation (Figure 2B) as measured by external aortic width expansion (mean width; saline—Cre0/0: 0.96 ± 0.03 mm vs. Cre+/0: 0.98 ± 0.01 mm, *p* = NS; AngII—Cre0/0: 1.87 ± 0.20 mm vs. Cre+/0: 1.26 ± 0.08 mm, *p* < 0.001; Two‐way ANOVA), which strongly demonstrates that adipocyte‐derived calpain‐2 plays a critical role in obesity‐accelerated AAA development in AngII‐infused mice. Furthermore, adipocyte‐specific calpain‐2 deficiency also significantly reduced AngII‐induced AAA incidence (Figure 2C; Cre0/0: 76% vs. Cre+/0: 22%, *p* < 0.001; Fisher exact test), but had no significant effect on aortic rupture (Cre0/0: 33% vs. Cre+/0: 18%). Verhoeff's staining of abdominal aortas showed preserved medial elastin layer (Figure 2D) in adipocyte‐specific calpain‐2–deficient mice (Cre+/0) upon AngII infusion, compared to littermate controls (Cre0/0) (elastin breaks; saline—Cre0/0: 1.25 ± 0.28 vs. Cre+/0: 1.0 ± 0.0, *p* = NS; AngII—Cre0/0: 6.3 ± 0.70 vs. Cre+/0: 2.75 ± 0.29, *p* < 0.05; Two‐way ANOVA). Furthermore, picrosirius red staining revealed intact adventitial collagen deposition in the adipocyte calpain‐2–deficient mice compared to Cre0/0 mice which showed disrupted adventitial collagen deposition (Figure 2E). Previously, we demonstrated that calpain inhibition suppressed leukocyte accumulation in the adipose tissue of diet‐induced obese mice [4]. Here, by immunohistochemical staining using anti‐CD45 antibodies, we identified that adipocyte‐specific calpain‐2 deficiency prevented leukocyte accumulation in the PAAT (Figure 2F) compared to the littermate controls (Cre0/0).

## 4 Discussion

Interestingly, the extent of protection against AngII‐induced AAA formation is equivalent in both ubiquitous and adipocyte‐calpain‐2–deficient obese mice. This observed protection could be due to the observed strong reduction of calpain‐2 in the PAAT in both global and adipocyte‐specific deficient mice. To our knowledge, this study is the first report to imply PAAT‐derived calpain‐2 in obesity‐accelerated AngII‐induced AAAs. These results suggest that the inhibition of calpain‐2 may offer a new therapeutic target to reduce AAAs in obese patients. However, currently it is not clear how calpain‐2 derived from adipocytes promotes AAA formation in obese mice. One possible mechanism could be due to defective exosome secretion from calpain‐2 adipocytes. In support, adipose tissue derived exosomes are shown to play a major role in promoting inflammation in obese patients/mice by acting as a carrier of cell secreted cargos including microRNAs and various cytokines [8, 9]. In addition, calpains are also shown to modulate microvesicles secretion by cleaving annexins [10].

Future studies are warranted to examine whether calpain‐2 mediates its effect either by directly promoting perivascular inflammation or indirectly by accelerating cytoskeletal structural protein destruction‐mediated aortic extracellular matrix protein degradation via periaortic secreted exosomes.

## Author Contributions

Conceptualization: V.S. and H.A.U. Implementation of animal experiments: A.C.F., A.J., M.O., W.J., and L.Y. Data analyses: A.C.F., A.J., and V.S. Supervising and data verification: V.S. Writing the draft manuscript: A.C.F., A.J., and V.S. Editing the manuscript: V.S. All authors have read and agreed to the published version of the manuscript.

## Conflicts of Interest

The authors declare no conflicts of interest.

## Data Availability

The data, analytic methods, and study materials will be maintained by the corresponding author and made available to other researchers upon reasonable request.
